# Role of Ubiquitin-Mediated Degradation System in Plant Biology

**DOI:** 10.3389/fpls.2016.00806

**Published:** 2016-06-08

**Authors:** Bhaskar Sharma, Deepti Joshi, Pawan K. Yadav, Aditya K. Gupta, Tarun K. Bhatt

**Affiliations:** Department of Biotechnology, School of Life Sciences, Central University of Rajasthan, Bandar SindriRajasthan, India

**Keywords:** ubiquitin, plant stress, plant immunity, E3 ligase, plant abiotic response

## Abstract

Ubiquitin-mediated proteasomal degradation is an important mechanism to control protein load in the cells. Ubiquitin binds to a protein on lysine residue and usually promotes its degradation through 26S proteasome system. Abnormal proteins and regulators of many processes, are targeted for degradation by the ubiquitin-proteasome system. It allows cells to maintain the response to cellular level signals and altered environmental conditions. The ubiquitin-mediated proteasomal degradation system plays a key role in the plant biology, including abiotic stress, immunity, and hormonal signaling by interfering with key components of these pathways. The involvement of the ubiquitin system in many vital processes led scientists to explore more about the ubiquitin machinery and most importantly its targets. In this review, we have summarized recent discoveries of the plant ubiquitin system and its involvement in critical processes of plant biology.

## Introduction

Involvement of the ubiquitin-proteasome system (UPS) at the cellular level has received great attention over the past decade. This particular topic attracted researchers more after ‘Nobel Prize’ in ‘Chemistry’ given for the discovery of ubiquitin-mediated protein degradation. UPS controls the degradation of many proteins in the cells and affects a range of cellular processes like signal transduction, cell division, immune responses and much more (Hershko, 2005). It controls the protein content of the cell, including essential enzymes such as kinases, phosphatases, and plant hormones involved in signaling and cellular regulatory pathways (**Table 1**) (Stone and Callis, 2007; Yang et al., 2010). In plants, ubiquitin-mediated degradation plays an important role in growth, hormonal signaling, abiotic stress, embryogenesis, and senescence. About 1600 genes (approximate 6% of the total genome) are involved in Ubiquitin/26S proteasome system and related functions in *Arabidopsis thaliana* (Mazzucotelli et al., 2006; Vierstra, 2009). Ubiquitin is a 76-amino acid long protein linked to lysine residues in target protein (Hershko et al., 2000). The process of ubiquitination is very complex where three enzymes are typically required, namely E1, E2, and E3 ligases. An E1 enzyme catalyzes the activation of UPS by adenylation of the C-terminal carboxyl group of ubiquitin (Schulman and Harper, 2009; Berndsen and Wolberger, 2014). The activated ubiquitin is then transferred from E1 to a cysteinyl residue of E2 ubiquitin conjugating enzyme followed by final transfer of ubiquitin to substrate mediated by an E3 ubiquitin ligase enzyme. The E3 ligases can ubiquitinate the substrate via either direct ubiquitin transfer or thioester formation with ubiquitin (Spratt et al., 2012). E3 ligase recognizes the substrate and form isopeptide bond between lysine residue of substrate and C-terminus of ubiquitin. Normally, isopeptide bond is formed with the 𝜀-NH2 group on substrate lysyl residue, but ester or thioester linkages were also seen in mammalian cells. The addition of more ubiquitin to the substrate or target is repeatedly done by the E3 ligase, required for substrate recognition by 26S proteasomal degradation system (Komander and Rape, 2012). However, E4 is also reported to be involved in the ubiquitination process in some species (Koegl et al., 1999; Smalle and Vierstra, 2004). The 26S proteasomal degradation system consists ofthe 19S regulatory particle (RP) and the 20S core protease. It is responsible for degradation of the target protein. The release of ubiquitin moiety of tagged protein is essential for recycling of ubiquitin to be used for the next round of ubiquitination and is done by (DUB) deubiquitinating enzyme (Turcu et al., 2009). Recent studies have discovered the various sites of ubiquitination on the substrate. The ubiquitination found on K63 (Lysine-63) linkages have been discovered in yeast and mammalian cells, and in plant it is involved in DNA replication, repair, protein synthesis, intracellular auxin level, and iron deficiency affecting root development (Jacobson et al., 2009; Li and Schmidt, 2010; Leitner et al., 2012). Typical connections of ubiquitin are via (K48) lysine-48 linkages (Dreher and Callis, 2007). The K29-linked chains have also been discovered which are involved in gibberellic acid responses via DELLA receptor degradation (Wang et al., 2009). Similarly, the ubiquitin chains linked via K6, K11, K27, and K33 were found in mammalian DNA repair and proteasomal degradation (Wu et al., 2008; Xu et al., 2009).

**Table 1:** List of E3 Ubiquitin ligase units and their targets involved in abiotic stress and plant immunity.

Ubiquitin ligase	Target	Role	Reference
KEG (the keep on going)	ABI5	Abscisic acid (ABA) signaling and abiotic stress response	Stone et al., 2006; Liu and Stone, 2013, 2014
ABD1 (the ABA-hypersensitive DCAF1)	ABI5	ABA signaling and abiotic stress response	Seo et al., 2014
CUL4-DDB1 E3 ligases DWD hypersensitive 1 and 2	ABI5	ABA signaling and abiotic stress response	Lee et al., 2010
AIP2 (RING E3 ligase ABI3-interacting protein 2)	ABI3	ABA signaling and abiotic stress response	Kurup et al., 2000
SDIR1	SDIRIP1 (SDIR1-Interacting Protein1)	Abiotic stress response	Zhang et al., 2015
Rma1H1	Aquaporin Isoform PIP2;1	Abiotic stress response	Lee et al., 2009
VuDRIP	VuDREB2A	Abiotic stress response	Sadhukhan et al., 2014
CUL4-DDB1	DDI1	Abiotic stress response	Miao et al., 2014
CUL4-DDB1-DET1	GLK2 (Golden 2-Like)	Abiotic Stress response	Tang et al., 2015
(SAUL1/plant U-box) AtPUB	AAO3	ABA-dependent stress response	Salt et al., 2011
AtRZF1	Proline content	Abiotic stress response	Ju et al., 2013
HOS1	ICE1	Abiotic stress response	Dong et al., 2006
COP1 (Constitutively Photomorphogenic 1)	UVR8	Abiotic stress response	Favory et al., 2009
MIEL1	MYB30	Plant immunity	Marino et al., 2013
SR1IP1	AtSR1	Plant immunity	Zhang et al., 2014
AvrPtoB	Fen	Plant immunity	Mathieu et al., 2014
XB3	XA21	Plant immunity	Huang et al., 2013
PUB12 and PUB13	FLS2	Plant immunity	Lu et al., 2011
EIRP1	VpWRKY11	Plant immunity	Stegmann et al., 2012
PUB22	Exo70B2	Plant immunity	Stegmann et al., 2012
Cullin 3-based E3 ligases	NPR1 (non-expresser of PR genes 1)	SA perception and signal transduction during the plant defense response	Fu et al., 2012

**FIGURE 1:**
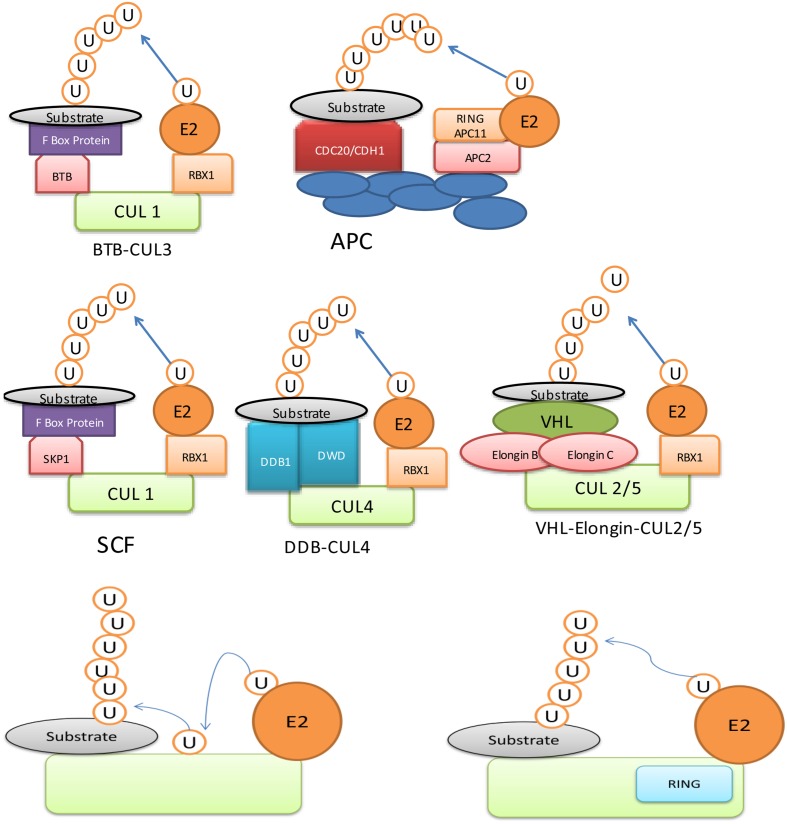
**Ubiquitin E3 ligase can be divided into two major types: **(i)** Homologous to E6-AP C-Terminus (HECT) domain containing E3, **(ii)** Really Interesting New Gene (RING) domain containing E3**.

Being a major determinant of ubiquitination process, E3 ligases have been studied in great detail. It contains RING (Really Interesting New Gene) domain, which binds to E2 enzyme and ubiquitinates the substrate via either monoubiquitination, multi-monoubiquitination or polyubiquitination (Sun and Chen, 2004; Deshaies and Joazeiro, 2009). Ubiquitination is also used as information signal apart from having a role in degradation. For example, monoubiquitin can act as a signal for DNA repair and vesicle trafficking, whereas poly-ubiquitin act as markers for protein kinase activity (Johnson, 2002). An E3 ubiquitin ligase may have different ubiquitin ligase domains such as ‘Homologous to E6-AP *C*-Terminus’ (HECT) (**Figure 1**) and ‘Really Interesting New Gene’ (RING)/U-box domain (**Figure 1**). The HECT domain is conserved region made up of 350-residues (Huibregtse et al., 1995) and it is a bi-lobed structure, which binds to E2 enzyme at N-terminal and ubiquitin through thioester linkage at the C-terminal (Huang et al., 1999). An E3 ligase, containing ‘RING’domain is further divided into two types: (i) single unit containing RING/U-box domain, which directly binds to substrate and (ii) multisubunit containing either RBX1 (ring-box 1) or APC11 (Anaphase Promoting Complex 11) (**Figure 1**). The multisubunit E3 ligases function in many complexes, which include SCF (SKP1-CULLIN-F-box), CUL3 (CULLIN 3)-BTB/POZ (Bric a brac, Tramtrack and Broad complex/Pox virus and Zinc finger), CUL4-DDB1 (UV-Damaged DNA Binding Protein 1) and APC (Anaphase Promoting Complex) in plants (Pickart, 2001; Lyzenga and Stone, 2012). These proteins recognize and ubiquitinate specific substrate to be processed by the proteasomal degradation system (Gagne et al., 2002; Zheng et al., 2002; Santner and Estelle, 2010; Wang and Deng, 2011; Sadanandom et al., 2012).

## Role of UPS in Abiotic Stress

Plants have a special capacity to grow and adapt themselves to environmental stress conditions like drought, salinity, radiation, heavy metals, and nutrient deprivation. Proteome present in a cell controls the stress condition (Smalle et al., 2003; Kurepa et al., 2008). The UPS has a vital role in altering protein load in the cell by degradation. This degradation of proteins affects many cellular activities including gene expression and signaling. There are complex signaling mechanisms that take place in plant cells during environmental stress conditions. A large number of instances reported in the literature where UPS is involved in regulating stress responses directly or indirectly. For example, E3 ubiquitin ligases are involved in regulating drought and salinity stress through Abscisic Acid (ABA) signaling. The transcription factors like ABI5 (ABA-insensitive *5*) and bZIP (basic leucine zipper) control many proteins of ABA signaling such as ABI3/4/5 (ABA-insensitive 3/4/5), ABF1/3 (ABA-Responsive Elements Binding Factor 1/3), and HB6 (Homeobox-Leucine zipper protein-6; Lopez-Molina et al., 2001). The ABI5 is regulated by some E3 ligases like KEG (the Keep On Going; Stone et al., 2006; Liu and Stone, 2013, 2014), ABD1 (the ABA-hypersensitive DCAF1; Seo et al., 2014), and the two CUL4-DDB1 E3 ligases DWD hypersensitive 1 and 2 (Lee et al., 2010). ABI3, a B3-type transcription factor participates in the desiccation tolerance and dormancy during zygotic embryogenesis (Lopez-Molina et al., 2001, 2002), interacts with the RING E3 ligase ABI3-interacting Protein 2 (AIP2) for proteasomal degradation (Kurup et al., 2000). In normal condition, KEG maintains low levels of ABI5 in the cytoplasm during ABA signaling (Stone et al., 2006; Liu and Stone, 2013). However, under stress conditions, ubiquitin-mediated degradation of the KEG takes place which results in accumulation of ABI5 (Liu and Stone, 2010). Similarly, CaAIR1 (*Capsicum annuum* ABA-Insensitive RING protein 1, hot pepper) gene is necessary for drought stress response. CaAIR1 contains a C3HC4 type RING finger motif, required for attachment of ubiquitins to the target protein. The considerable changes in the stomata and regulation of gene expression during stress due to CaAIR1 suggesting its negative role in drought resistance (Park et al., 2015). Another example of a C3HC4 RING finger E3 ligase is OsDIS1 (*Oryza sativa* drought-induced SINA protein 1) which is involved in drought stress regulation in rice. It contains a conserved region of RING domain, responsible for ubiquitination. The expression of *OsDIS1* was up-regulated under drought conditions. It is shown that OsDIS1 plays a negative role by altering the transcriptional regulation of stress-related genes and possibly by regulating post-translational events in OsNek6 protein (*O. sativa* NIMA-related kinase 6; Ning et al., 2011). A recent work on the intracellular E3 ligase SDIR1 (Salt- and Drought-Induced Really interesting new gene finger1) proved its vital role in abiotic stress and ABA signaling. SDIR1 interacts with its substrate SDIRIP1 (SDIR1-Interacting Protein1) and alters the SDIRIP1 stability by Ubiquitin/26S proteasome pathway. SDIRIP1 regulates the ABA-Insensitive 5 expression via a leucine-zipper transcription factor rather than ABA-responsive Elements Binding Factor 3 (ABF3) or ABF4, to regulate ABA-mediated seed germination and the plant salt response. Therefore, SDIR1 positively regulates the ABA signaling and abiotic stress response (Zhang et al., 2015). Transgenic plants with mutant drip1 and drip2 showed the increased gene expression suggesting the negative role of DRIP genes in drought stress (Qin et al., 2008). The over-expression of a hot pepper (*C. annuum*) homolog of a human RING membrane-anchor 1 E3 ubiquitin ligase, Rma1H1, in transgenic *A. thaliana* plants conferred enhanced stress tolerance. A novel membrane-bound E3 ubiquitin ligase gene BnTR1 isolated from *Brassica napus* regulates calcium channels and expression of heat shock proteins in the plant, therefore, contributing thermal resistance to plants (Liu et al., 2014).

The list of stress-related genes regulated by ubiquitination is very long. The trafficking of an aquaporin isoform PIP2;1 from ER to the plasma membrane was inhibited by Rma1H1 over-expression. The function of Rma1H1 was interfered by MG132, an inhibitor of the 26S proteasome, showing that Rma1H1 is involved in proteasomal degradation in abiotic stress condition by regulating aquaporin levels in plants (Lee et al., 2009). Further, adding to the list, AtAIRP1, 2 and 4 (*Arabidopsis* ABA-insensitive RING protein 1, 2, and 4), a C3H2C3-type RING (Really Interesting New Gene) E3 ubiquitin ligase, are positive regulators in the *A. thaliana* ABA-dependent drought response (Cho et al., 2011; Yang et al., 2015). The cowpea RING ubiquitin ligase VuDRIP binds to transcription factor VuDREB2A and regulates abiotic stress response. In normal condition, the transcription factor expression is low, but under desiccation, its expression is high. The Ubiquitin-mediated degradation of VuDREBA2A in non-stressed condition is suggested in cowpea and *Arabidopsis* (Sadhukhan et al., 2014). A Tomato DDI1 gene is responsible for the multiple abiotic stress responses such as against UV-C, high salinity, and mannitol. CUL4-DDB1 based ubiquitin ligase binds the DDI1 protein and regulates the abiotic stress via nucleus-dependent regulation of signaling for DNA damage repair, salt, and osmotic stress response in tomato (Miao et al., 2014). DDA1 (*A. thaliana* DET1-DDB1-ASSOCIATED1), part of COP10-DET1-DDB1 (CDD)-related complexes, negatively regulates ABA mediated developmental processes by interacting the ABA receptor PYL8, PYL4, and PYL9 (Irigoyen et al., 2014). A recent report on GOLDEN 2-LIKE (GLK2) transcription factor in tomato, degraded by CUL4-DDB1-DET1 ubiquitin ligase complex determined by two lysine residues K11 and K253 (Tang et al., 2015). The proteasomal degradation of *Arabidopsis* aldehyde oxidase 3 (AAO3), an ABA inducer, by a U-box type E3 senescence-associated E3 ubiquitin ligase 1 (SAUL1)/plant U-box (AtPUB) strongly suggests its involvement in abiotic stress mediated by ABA signaling (Salt et al., 2011). A RING E3 ligase, HOS1 negatively regulates the plant cold response by degrading transcription factor ICE1 (Inducer of CBF expression 1), an MYC-like basic helix–loop–helix transcription activator (Dong et al., 2006). Another example of negative regulation of abiotic stress is RING zinc finger 1 in *A. thaliana* (AtRZF1). A high concentration of proline was accumulated in AtRZF1 mutant plants, whereas overexpression of AtRZF1 conferred high sensitivity to drought stress (Ju et al., 2013). The plant UV-B perception system is also targeted by the ubiquitin system. UVR8 (UV Resistance Locus 8), a positive regulator of UV-B response, interacts with the RING finger E3 ligase, COP1 (Constitutively Photomorphogenic 1), thereby regulating UV-B response (Favory et al., 2009).

There are many more proteins such as F-box protein, Drought tolerance repressor (DOR), Ethylene Response Factor (ERF53), SaSce9 (*Spartina alterniflora* SUMO conjugating enzyme 9), R2R3 MYB transcription factor, *Botrytis* Susceptible1 (BOS1) and 9-*cis*-epoxycarotenoid dioxygenase 3(NCED3) are regulated by proteasomal degradation through ABA response to stress. Taken together, all these studies showed that UPS plays a vital role in abiotic stress conditions. There is a complex and coordinated network of processes for ubiquitin-mediated degradation of proteins involved in stress response through hormone signaling. Therefore, there is a scope of identifying specific signals required for combating stress responses in plants out of general proteasomal degradation of cellular proteins.

## Role of UPS in Plant Immunity

Plants are constantly exposed to potentially pathogenic microbes present in its surrounding environment. The pattern-triggered immunity (PTI) in plants is a vital mechanism of protection against pathogenic microorganisms. Detection of pathogen-associated molecular patterns (PAMPs) by pattern-recognition receptors (PRRs), is crucial for combating potential pathogens and these PRR complexes are in turn controlled by E3 ligases (Monaghan and Zipfel, 2012; Macho and Zipfel, 2014). A large number of reports authenticates the role of the proteasomal system in the plant defense/immune mechanism. A positive regulation of hypersensitive cell death mechanism in *Arabidopsis* was noticed by MYB30 transcription factor. However, MIEL1 (MYB30-Interacting E3 Ligase1), an *Arabidopsis* RING E3 ubiquitin ligase ubiquitinates and degrades the MYB30 by proteasomal degradation pathway and thus controls the hypersensitive cell death in *Arabidopsis* (Marino et al., 2013). The calcium ions play very important role in intracellular signaling as its concentration influences the plant immunity. The CaM-binding transcription factor AtSR1 involved in the repression of the EDS1 (Enhanced Disease Susceptibility 1) at the transcriptional level of calcium-mediated signaling (Du et al., 2009), is degraded by AtSR1 interaction protein 1 (SR1IP1) using proteasomal system; this is an example of positive regulation of plant immunity (Zhang et al., 2014). Salicylic acid is a plant immune signal which induces systemic acquired resistance. A transcription cofactor non-expresser of PR genes 1 (NPR1), required for systemic acquired resistance, is targeted by Cullin 3 ubiquitin E3 ligase thereby, interfering response to pathogen challenge (Fu et al., 2012). Citing further examples of plant immunity regulation via ubiquitination by introducing Pto and Fen kinases that activate effector-triggered immunity (ETI). The AvrPtoB, the *Pst* virulence protein, contains an E3 ubiquitin ligase domain, which causes degradation of Fen and reduces its ability to activate ETI. The proximity of the Pto kinase determines whether it will trigger an immunity response or will be degraded by E3 ligase domain of AvrPtoB. The binding of Pto kinase to the distal region of AvrPtoB evade the degradation, allowing ETI response to occur, whereas binding near to E3 ligase domain causes degradation of Pto (Mathieu et al., 2014). Also, microbial E3 ligase (*Pseudomonas syringae*) AvrPtoB effector shows unpredicted homology with U-box and RING-finger components of eukaryotic E3 ubiquitin ligases and therefore, microbial E3 mimics host E3 ubiquitin ligases to inactivate plant immunity (Janjusevic et al., 2006). Recently, a link between disease resistance in plants and cell cycle was proved. Overexpression of *Arabidopsis* gene OSD1 (Omission of the Second Division) and its homolog UVI4 (UV-B-Insensitive 4) negatively regulates anaphase-promoting complex/cyclosome (APC/C), a multi-subunit ubiquitin E3 ligase that regulates the progression of cell cycles (Bao et al., 2013). Interestingly, altering programmed cell death also induces plant disease resistance. The receptor kinase XA21 provides resistance to bacterial blight disease of rice (*O. sativa*) caused by *Xanthomonas oryzae pv. Oryzae* (Xoo), which is regulated by XA21 binding protein 3 (XB3), a RING finger-containing E3 ubiquitin ligase known for having a positive impact in XA21-mediated resistance (Huang et al., 2013). To add further, *Arabidopsis* PRR Flagellin-Sensing 2 (FLS2), interact with BAK1 and initiates immune signaling. The flagellin recruits two U-box E3 ubiquitin ligases, PUB12, and PUB13, to FLS2 receptor complex. PUB12 and PUB13 polyubiquitinate FLS2 receptor followed by flagellin-induced degradation attenuates the immunity in *Arabidopsis* (Lu et al., 2011). Likewise, there are several studies confirming the role of ubiquitin system in altering plant immune response such as a F-box protein CPR1/CPR30 as a negative regulator of an R protein SNC1 through SCF (Skp1-cullin-F-box) mediated protein degradation (Gou et al., 2012), role of E3 ubiquitin ligase Erysiphenecator Induced RING finger Protein 1 (EIRP1) in the defense response of Chinese wild grapevine by degrading VpWRKY11 transcription factor, PUB22 protein which degrades Exo70B2 by 26S proteasome degradation to contribute in attenuation of PAMP-triggered response (Stegmann et al., 2012) and avirulence effector AvrPiz-t from the rice blast fungus *Magnaporthe oryzae* repressing PAMP-triggered immunity in rice by suppressing rice RING E3 ubiquitin ligase APIP6. Therefore, over the past few years the role of ubiquitin-proteasomal degradation pathway in plant immune response has been explored intensively and hence established UPS as an integral part of cellular activities and critical for adapting immunity by plant cells. Future directions toward understanding UPS in plant immunity will definitely help to develop valuable solutions to crop loss and low productivity.

## Conclusion

The impact of ubiquitin-mediated degradation of proteins in the cellular system determines many aspects of responses to external and internal stimuli. UPS has been identified as a powerful degradation mechanism controling growth, response and immunity in plants. UPS has characteristics of regulating substrate degradation either negatively or positively with time and temperature indicating its strong integration in the molecular mechanisms. Though, it is a challenge to fully characterize the UPS and its substrates for their diverse role in cellular metabolism; more structural and functional insights are needed to fully explore UPS and its role in various critical pathways of the cell such as abiotic stress, defense system, and various hormonal regulations. It would develop an improved utilization of resources for the better growth of plants.

## Author Contributions

BS, DJ, and PKY collected data and BS, AKG, and TKB wrote the manuscript.

## Conflict of Interest Statement

The authors declare that the research was conducted in the absence of any commercial or financial relationships that could be construed as a potential conflict of interest.
